# Incidence and Risk Factors of Nasal Pressure Injuries in Neonates Receiving Noninvasive Ventilation

**DOI:** 10.3390/jcm15020615

**Published:** 2026-01-12

**Authors:** Blgeis Elgadra, Lina Abdullah, Hafsa Alsharif, Abdelrahman Dirar, Janet Estalilla, Quennie Fernandes, Habeebah Fazlullah, Jojo Furigay, Roderick Pedron, Bilal Kanth, Mohammad A. A. Bayoumi, Ashraf Gad

**Affiliations:** 1 Department of Medical Education, Sidra Medicine, Doha P.O. Box 26999, Qatar; belgadra@sidra.org; 2 Department of Medical Education, Hamad Medical Corporation (HMC), Doha P.O. Box 3050, Qatar; leeno810@gmail.com (L.A.); hafsa.alsharif@cw.bc.ca (H.A.); 3 Department of Critical Care Medicine, Department of Pediatrics, University of British Columbia, Vancouver, BC V6T 1Z4, Canada; 4 Neonatal Intensive Care Unit (NICU), Women’s Wellness and Research Center (WWRC), Hamad Medical Corporation (HMC), Doha P.O. Box 3050, Qatar; aabdelrahim1@hamad.qa (A.D.); jestalilla@hamad.qa (J.E.); qfernandes1@hamad.qa (Q.F.); hfazlullah@hamad.qa (H.F.); jfurigay@hamad.qa (J.F.); rperdon@hamad.qa (R.P.); bkanth@hamad.qa (B.K.); moh.abdelwahab@hotmail.com (M.A.A.B.); 5 Weill-Cornell Medicine-Qatar, Doha P.O. Box 24144, Qatar

**Keywords:** nasal pressure injury, non-invasive ventilation, neonate, newborn, neonatal intensive care unit, Qatar, respiratory support, nasal trauma, nasal septal injury, neonatal resuscitation

## Abstract

**Background/Objective:** Nasal pressure injuries following non-invasive ventilation (NIV) have remained a common complication. Available evidence on injury severity characteristics, timing, and predictors of progression to moderate–severe injury, especially in large cohorts, is limited. The objective was to assess the incidence, characteristics and risk factors for nasal pressure injuries among neonates on NIV in a large tertiary neonatal intensive care unit (NICU). **Methods:** This retrospective observational study recruited all infants who experienced nasal pressure injury while on NIV from March 2018 to November 2022. The severity of the injury was categorized by the Fischer classification. Demographics, perinatal, respiratory, and device-related factors were examined. Multivariable logistic regression revealed independent predictors of moderate to severe injury. **Results:** There were 237 nasal injury episodes in 226 infants (0.406 per 100 device-days), considering 17,004 NICU admissions and 58,363 NIV device-days. Most injuries were mild (Stage I 81%) while 19% were moderate–severe (Stage II–III). Early injuries (≤3 days after NIV) were present in 83.5% of patients and were often related to the nasal bridge. In particular, late-onset injuries (>3 days) were more likely in infants with previous injury, exposure to postnatal steroids, longer prior intubation, or septal involvement. Moreover, multivariable analysis identified three specific independent predictors of moderate–severe injury previous nasal injury (aOR 6.25, 95% CI 1.11–35.35), septal or combined bridge/septum involvement (aOR 2.98, 95% CI 1.04–8.43), and prolonged period of positive pressure ventilation at birth (aOR 1.23 per minute, 95% CI 1.04–1.45). **Conclusions:** Most nasal pressure injuries seen during NIV are mild and early; however, recurrence, septal involvement, and prolonged resuscitative ventilation markedly increase the risk of severe injury. Improving surveillance on early NIV use, monitoring of septal pressure points, and proactive interventions with interface management will aid in minimizing preventable nasal morbidity.

## 1. Introduction

Noninvasive ventilation (NIV), including nasal continuous positive airway pressure (CPAP) and nasal intermittent positive pressure ventilation (NIPPV), is a cornerstone of respiratory support in preterm and critically ill neonates [1,2,3,4]. These modalities reduce the need for intubation and mechanical ventilation, thereby minimizing risks of ventilator-associated complications such as bronchopulmonary dysplasia and ventilator-associated pneumonia [2,5,6]. However, despite their clinical benefits, NIV interfaces are not without complications. One of the most frequent and under-recognized adverse effects is nasal pressure injury, which may result in pain, tissue necrosis, scarring, and long-term nasal deformity [7,8,9,10,11].

The reported incidence of NIV-related nasal injury in neonates varies widely, ranging from 20% to 60%, depending on population, device type, and surveillance methods. Most studies originate from single centers with small cohorts, and differences in staging systems and injury definitions complicate comparisons across settings. Furthermore, while risk factors such as low gestational age (GA), prolonged NIV use, and interface type have been described, evidence regarding predictors of more severe or recurrent injuries remains limited [7,10,12,13,14,15,16,17,18,19,20,21,22].

Understanding the incidence and risk profile of nasal injuries is essential for guiding preventive strategies, optimizing interface selection, and informing nursing protocols for device surveillance and skin care. Data from large cohorts in high-volume neonatal intensive care units (NICUs) are particularly valuable for identifying clinically significant patterns.

This study aimed to determine the incidence, severity distribution, and risk factors for nasal pressure injuries among neonates receiving NIV in a tertiary referral NICU over five years. In particular, we examined predictors of moderate–severe injury and the timing of injury onset, to identify infants at highest risk for adverse outcomes.

In addition, this work seeks to provide clinically applicable data that may inform interface selection, surveillance practices, and targeted preventive strategies in high-risk neonatal populations, while highlighting areas where prospective studies and standardized protocols are needed to reduce NIV-associated skin injury.

## 2. Materials and Methods

### 2.1. Design, Population, and Study Setting

This single-center, retrospective observational study was conducted at the Women’s Wellness and Research Center (WWRC), formerly known as the Women’s Hospital, in Doha, Qatar. Ethical approval was obtained from the WWRC Medical Research Center (MRC)/Institutional Review Board (IRB) under protocol number MRC-01-22-757. Between March 2018 and November 2022, our hospital recorded 17,004 NICU admissions. During the study period, NIV was delivered using commercially available neonatal masks and binasal prongs, selected according to unit protocol and infant size, with routine use of protective barriers when indicated. All nasal interfaces during the study period were manufactured by Fisher & Paykel Healthcare. Nasal mask sizes included Small, Medium, Large and Extra Large. Binasal prongs were available in four sizes (3020, 3520, 4030, and 5040), with size selection guided by manufacturer recommendations and routine clinical assessment. Following that, 237 episodes of nasal injury were identified in 226 infants, including 12 with recurrent injuries.

### 2.2. Inclusion and Exclusion Criteria

The study included all live-born infants admitted to the NICU who developed a nasal injury while receiving non-invasive respiratory support (NIPPV, high-flow nasal cannula, or CPAP) at any point during the study period. Demographic variables collected included gestational age, sex, and birth weight. Additional clinical variables included date of birth, duration and type of noninvasive respiratory support, nasal interface used, injury location (septum/bridge), and injury stage.

Infants with facial malformations, congenital anomalies, or facial birth trauma were excluded.

### 2.3. Injury Detection Method

Nasal pressure injuries in the neonatal population have been categorized according to the classification proposed by Fischer et al. [9]. Stage I: Non-blanching erythema on otherwise intact skin. The skin is red, but when pressure is applied, the redness does not disappear; Stage II: Partial loss of dermis thickness (superficial ulcer or erosion), presenting as a superficial, red wound with no crust; Stage III: Necrosis and total tissue loss, resulting in full-thickness loss of skin.

Nasal injury in neonates on non-invasive ventilation was detected through regular, structured skin assessments using a standardized tool, performed by the respiratory therapists every 4 h in collaboration with nursing staff and more frequently if clinically indicated. Detection relies on checking skin integrity, ensuring correct interface size and secure placement, confirming adequate humidification and the presence of a barrier, and promptly identifying any breakdown or early signs of injury. Staff must classify the injury using a uniform grading system [9], report abnormalities immediately to the physician and charge nurse, and document findings during multidisciplinary rounds.

When alternating interfaces were used, this referred to planned rotation between nasal mask and nasal prongs as part of routine skin-protection practice; every 4 h, or more or less, following the unit protocol and clinical judgement.

### 2.4. Objectives

The primary objectives of this study were to determine the incidence of nasal injury among infants receiving non-invasive respiratory support and to identify associated risk factors in the largest tertiary NICU in Qatar.

### 2.5. Data Collection

Data were extracted from maternal and neonatal medical records. Maternal variables included antenatal steroid exposure, clinical chorioamnionitis, and mode of delivery. Neonatal variables included date of birth, sex, nationality, GA, BW, plurality, birth-related facial trauma, Apgar scores at 1 and 5 min, and details of positive pressure ventilation (PPV) at birth (use and duration). Additional data included postnatal steroid or vasopressor use; type and settings of respiratory support at the time of injury (positive end-expiratory pressure (PEEP), fraction of inspired oxygen (FiO_2_)); hemoglobin, lactate, and base excess/deficit at injury onset; timing and duration of intubation; initiation date of non-invasive support; date and site of nasal injury (bridge/septum); interface type at injury; and injury severity.

For infants receiving NIV, the specific interface category (mask, prongs, or alternating) and the interface size selected according to manufacturer recommendations were recorded at the time of injury. Device sizing and fit were routinely assessed as part of standard respiratory therapy and nursing practice. Inappropriate interface size or fit was evaluated descriptively as a potential contributing factor but was not independently analyzed as a risk factor due to the inconsistent retrospective documentation.

### 2.6. Statistical Analysis

Descriptive analyses were conducted to evaluate patient characteristics and clinical variables. Continuous variables were summarized as mean and standard deviation (SD) for parametric data, and as median and interquartile range (IQR) for non-parametric data. Comparisons of continuous variables were made using Student’s *t*-test for parametric distributions or the Mann–Whitney U test for non-parametric distributions, as appropriate. Categorical variables were presented as frequencies and percentages, and compared using chi-square or Fisher’s exact tests. Multivariate logistic regression analyses were conducted to identify predictors associated with higher stages of nasal injury. Variables with *p*-values < 0.1 in univariate analyses were included in the multivariate model. All *p*-values were two-tailed, and values below 0.05 were considered statistically significant. Statistical analyses were conducted using SPSS software version 30 (IBM Corp., Armonk, NY, USA).

## 3. Results

During the study period, a total of 17,004 infants were admitted to the NICU. Across 2018–2022, there were 58,363 NIV device days recorded. In this cohort, 237 episodes of nasal injury were identified, corresponding to 0.406 per 100 device days (≈4 per 1000 device days).

A total of 237 nasal injury episodes were documented in 226 infants. Twelve infants experienced repeated injuries, and among them, three progressed from Stage I (mild) to Stage II (moderate) injury. As summarized in Table 1, of all episodes, 192 (81.0%) were classified as mild (Stage I) and 45 (19.0%) as moderate/severe (Stages II–III). Infants with moderate/severe injury required a longer duration of PPV at delivery (median 3 vs. 2 min, *p* = 0.033). GA and BW were slightly lower in the moderate/severe group, although these differences were not statistically significant. A low Apgar score at 1 min (<7) was significantly more common in the moderate/severe group compared with the mild group (68.9% vs. 48.4%, *p* = 0.013), while 5 min Apgar scores did not differ. No significant differences were observed in mode of delivery, chorioamnionitis, multiple gestation, sex distribution, small for gestational age status, or antenatal steroid exposure. Similarly, subgroup analyses by GA, BW, and growth categories showed no significant differences between groups.

Table 2 presents the clinical characteristics of infants with nasal injuries. Infants with moderate–severe injury had longer intubation durations before injury (median 1 vs. 0 days, *p* = 0.026). They were more likely to have had a previous injury (11.1% vs. 3.1%, *p* = 0.022), to have been on NIV before intubation (17.8% vs. 7.8%, *p* = 0.042), and to sustain injury on the septum or both bridge/septum (46.7% vs. 22.0%, *p* = 0.002). They were also more frequently injured when using prongs or alternating interfaces (22.2% vs. 8.4%, *p* = 0.017). Although mask use was more common overall, the proportion of moderate-severe injury was higher among infants exposed to prongs or alternating interfaces, indicating a relatively increased risk compared with mask-only use. Other parameters, including FiO_2_, lactate, hemoglobin, base deficit, and diagnosis, did not differ significantly between groups. Timing analysis showed that injuries occurring after ≥3 weeks were significantly more likely to be moderate–severe (24.4% vs. 10.4%, *p* = 0.037), and injuries developing beyond the first 3 days of life tended to be more severe (48.9% vs. 33.3%, *p* = 0.051).

In the multivariable logistic regression analysis (Table 3) predicting moderate–severe nasal injuries, several candidate variables were entered into the model, including injury location (bridge, septum, or both), interface type (mask, prongs, alternating), previous injury, duration of PPV at delivery, PEEP at the time of injury, Apgar scores at 1 and 5 min, hemoglobin level at injury, prior use of NIV before intubation, timing of injury (day of life), and days on NIV before injury. After stepwise forward selection, three factors remained significant independent predictors of moderate–severe nasal injury. Infants with a previous nasal injury had a more than six-fold increased risk of developing moderate–severe injury (aOR 6.25, 95% CI 1.11–35.35, *p* = 0.038). Injury location at the septum or involving both bridge and septum was also associated with a nearly three-fold higher risk compared to bridge-only injuries (aOR 2.98, 95% CI 1.04–8.43, *p* = 0.043). In addition, each additional minute of PPV at delivery was associated with a 23% increase in the odds of moderate–severe nasal injury (aOR 1.23, 95% CI 1.04–1.45, *p* = 0.014).

Among 237 infants with nasal injury, 169 (71.3%) developed injury within ≤3 days of NIV initiation (Table 4), while 68 (28.7%) developed injury after >3 days. Late-onset injuries were significantly associated with prior nasal injury (14.7% vs. 0.6%, OR 28.97, *p* < 0.001), exposure to postnatal steroids before injury (10.3% vs. 0.6%, OR 19.3, *p* < 0.001), lower FiO_2_ at injury (0.24 vs. 0.27, *p* = 0.006) with fewer requiring FiO_2_ ≥ 0.30 (6.0% vs. 18.9%, OR 0.27, *p* = 0.013), lower hemoglobin (13.7 vs. 15.7 g/dL, *p* < 0.001), lower lactate (1.1 vs. 1.5 mmol/L, *p* < 0.001), and less pronounced base deficit (0.16 vs. −2.41, *p* < 0.001). These infants also had a longer duration of intubation before injury (*p* < 0.001), more days on NIV before injury (9.5 vs. 1.3 days, *p* < 0.001), and a later onset of injury (16.8 vs. 3.3 days of life, *p* < 0.001).

Injury location differed, with late-onset cases more likely to involve the septum/columella (41.2% vs. 16.0%) compared to bridge injuries (55.9% vs. 79.9%, *p* < 0.001). No significant differences were observed in the type of respiratory support at injury, PPV at birth, vasopressor use, severity stage, PEEP levels, or primary respiratory diagnosis. The timing of onset varied, as nearly 90% of early injuries occurred within the first week of life, whereas late-onset injuries frequently presented after day 7, including 36.8% after 14 days (*p* < 0.001).

These findings indicate that interface-related factors and patient-related factors differ substantially between early and late injury phenotypes.

## 4. Discussion

To our knowledge, this study presents one of the largest single-center datasets describing nasal pressure injuries in neonates receiving non-invasive ventilation. With a large number of NICU admissions and device-use days, the overall incidence of nasal injury in our population was low compared with the higher rates commonly reported in smaller studies [12,13,14,15,16,17,18,19,20,21,22]. Despite this lower incidence, the findings confirm that nasal pressure injury remains a relevant clinical problem [9], showing clear differences between injuries that appear early versus those that develop later, and between mild and more advanced stages [8].

Our hospital applies a strict preventive skin assessment tool which prioritizes safe interface application measures (e.g., masks preferred to prongs, barriers, humidification, regular checking of interface size and placement, rotation of devices) [23,24].

Most injuries developed soon after NIV was initiated, with the first few days representing the period of greatest risk [7,8,9]. Infants with early injuries showed signs of greater illness severity, including higher oxygen needs and biochemical markers suggesting reduced tissue perfusion. These injuries tended to involve the nasal bridge, an area more exposed to interface pressure [25].

Late-onset injuries showed a very different profile. They were more often seen in infants who had previously sustained a nasal injury, had received postnatal steroids, or had longer periods of intubation before starting non-invasive support [25,26,27]. This pattern points to accumulated tissue fragility and impaired healing as contributors. Late injuries also involved the nasal septum more frequently, an area known to have thin epidermis, end-arterial blood supply and a higher risk of deterioration [10,28].

When examining injury severity, several clinical factors stood out. Infants developing moderate to severe injuries were more likely to have required longer positive pressure ventilation at birth and to have had lower Apgar scores, reflecting the impact of perinatal instability on later tissue vulnerability. Longer intubation before the onset of injury also contributed to severity [29]. Anatomical location played an important role as well; injuries involving the septum (or affecting both the septum and nasal bridge) were substantially more likely to become moderate to severe [28]. Interface type influenced outcomes too, with prongs or alternating interfaces showing a higher tendency toward more advanced injury compared with mask use alone [10,21]. This finding should be interpreted in the context of overall interface exposure, as masks were used more frequently in routine care; however, when injuries occurred during prong or alternating use, they were more likely to be of higher severity. Injuries that occurred after a more prolonged course of non-invasive ventilation were also more likely to be severe, suggesting a cumulative pressure effect [9,22].

Multivariable analysis identified three independent predictors of moderate to severe injury: a previous nasal injury, involvement of the septum, and the need for longer resuscitative ventilation at birth. These predictors highlight the need for increased vigilance in neonates with recurring injuries, septal involvement, or those who experienced more intensive support immediately after birth.

Clinically, these findings underscore several key points:

1. The early days of NIV are crucial, requiring consistent and structured skin assessments [10,11,30].
2. A history of previous injury should be treated as a marker of vulnerability, prompting measures such as interface rotation and protective barriers [23,24].
3. Septal injuries deserve particular attention due to their association with more severe outcomes.
4. Perinatal resuscitation appears to play a role in later injury severity, suggesting that infants with difficult transitions at birth may benefit from closer monitoring.
5. Finally, interface selection matters: while prongs may be necessary in some clinical situations, careful alternation with masks and appropriate skin protection may help reduce severity.

### Strengths and Limitations

The large sample size, systematic staging of injuries, and multivariable analysis are strengths of this study. Limitations include its retrospective design, potential underreporting of very mild or transient injuries, and the single-center setting, which may limit generalizability due to variations in device type, nursing practices, and skin care protocols. Long-term outcomes such as nasal deformity and cosmetic sequelae were not assessed and should be explored in future prospective studies. Late-onset injuries may also be biassed toward infants who survived long enough or required prolonged NIV, potentially inflating risk associations. Future research should include prospective evaluation of specific NIV device brands, interface sizing accuracy, and standardized interface-rotation schedules to better define modifiable risk factors for nasal injury.

## 5. Conclusions

Most NIV-related nasal injuries in neonates are mild and occur early, but recurrence, septal involvement, and prolonged PPV at delivery substantially increase the risk of progression to moderate–severe injury. Vigilant monitoring during the first days of NIV, early recognition of septal involvement, and proactive interface management are critical to preventing severe complications.

## Figures and Tables

**Table 1:** Baseline Characteristics of Infants with Mild (Stage I) versus Moderate–Severe (Stage II–III) Nasal Injury.

Variable	Mild (n = 192)	Moderate–Severe (n = 45)	*t*/Z/χ^2^	*p*-Value
Gestational Age (weeks)	28.24 ± 3.46	27.69 ± 3.44	0.97	0.166
Birth Weight (g)	1240.81 ± 583.19	1163.78 ± 444.02	0.83	0.203
Mode of delivery				
NVD	69 (35.9%)	20 (44.4%)	1.13	0.289
LSCS	123 (64.1%)	25 (55.6%)		
Chorioamnionitis				
No	183 (95.3%)	43 (95.6%)	0.01	0.944
Yes	9 (4.7%)	2 (4.4%)		
Multiple gestation				
No	134 (69.8%)	34 (75.6%)	0.59	0.444
Yes	58 (30.2%)	11 (24.4%)		
Gender				
Male	106 (55.2%)	26 (57.8%)	0.1	0.755
Female	86 (44.8%)	19 (42.2%)		
Duration of PPV at delivery (min)	2 (1–4)	3 (2–5)	−2.132	0.033
Apgar 1 < 7	97 (51.6%)	14 (31.1%)	6.11	0.013
Apgar 5 < 7	15 (7.9%)	6 (13.3%)	1.3	0.255
Antenatal steroids				
No	42 (21.9%)	9 (20.0%)	0.08	0.783
Yes	150 (78.1%)	36 (80.0%)		
PPV at birth				
No	86 (44.8%)	14 (31.1%)	2.8	0.094
Yes	106 (55.2%)	31 (68.9%)		
GA categories				
<28 weeks	100 (52.1%)	26 (57.8%)	0.717	0.699
28–32 weeks	59 (30.7%)	11 (24.4%)		
>32 weeks	33 (17.2%)	8 (17.8%)		
Birth weight categories				
<1000 g	83 (43.2%)	17 (37.8%)	1.112	0.573
1000–1500 g	61 (31.8%)	18 (40.0%)		
>1500 g	48 (25.0%)	10 (22.2%)		
Growth category				
AGA	172 (89.6%)	38 (84.4%)	0.976	0.614
LGA	12 (6.3%)	4 (8.9%)		
SGA	8 (4.2%)	3 (6.7%)		

Values are presented as mean ± SD, median (IQR), or n (%). Statistical comparisons were performed using Student’s *t*-test, Mann–Whitney U test, or χ^2^ test, as appropriate. Abbreviations: PPV: positive pressure ventilation; NVD: normal vaginal delivery; CS: caesarean section; GA: gestational age; SGA: small for gestational age; AGA: appropriate for gestational age; LGA: large for gestational age.

**Table 2:** Clinical characteristics of infants with mild versus moderate–severe nasal injury.

Variable	Mild (n = 192)	Moderate–Severe (n = 45)	*t*/Z/χ^2^	*p*-Value
PEEP (cmH_2_O)	6 (6–7)	6 (6–7)	−1.588	0.112
Lactate (mmol/L)	1.3 (1.0–1.8)	1.4 (1.0–1.75)	−0.431	0.666
FiO_2_	0.262 ± 0.071	0.276 ± 0.103	−1.11	0.135
Hemoglobin (g/dL)	15.30 ± 3.08	14.41 ± 2.77	1.77	0.078
Base deficit/excess	−1.82 ± 3.53	−1.10 ± 3.77	−1.22	0.224
NIV start (day of life)	3.28 ± 8.44	4.53 ± 7.09	−0.92	0.359
Age at injury (day of life)	6.80 ± 12.57	8.67 ± 9.39	−0.94	0.35
Injury timing (days on NIV)	3.54 ± 5.95	4.17 ± 4.76	−0.66	0.513
Early vs. late injury (days of life)			3.82	0.051
First 3 days	128 (66.7%)	23 (51.1%)		
>3 days	64 (33.3%)	22 (48.9%)		
Previous injury			5.25	0.022
No	186 (96.9%)	40 (88.9%)		
Yes	6 (3.1%)	5 (11.1%)		
Postnatal steroids before injury			0.23	0.634
No	185 (96.4%)	44 (97.8%)		
Yes	7 (3.6%)	1 (2.2%)		
Duration of postnatal steroids before injury (days)	9 (8–14)	14 (14–14)	−0.883	0.377
Vasopressor use before injury			0.1	0.572
No	189 (98.4%)	44 (97.8%)		
Yes	3 (1.6%)	1 (2.2%)		
Duration of intubation if intubated before injury (days)	0.00 (0–2.0)	1 (0–6.0)	−2.225	0.026
On NIV before intubation			4.13	0.042
No	177 (92.2%)	37 (82.2%)		
Yes	15 (7.8%)	8 (17.8%)		
Injury location			12.98	0.002
Bridge	149 (78.0%)	24 (53.3%)		
Septum	38 (19.9%)	17 (37.8%)		
Both	4 (2.1%)	4 (8.9%)		
Interface type			8.12	0.017
Mask	175 (91.6%)	35 (77.8%)		
Prongs	12 (6.3%)	6 (13.3%)		
Alternating	4 (2.1%)	4 (8.9%)		
NIV mode			3.11	0.211
NIPPV	133 (69.3%)	37 (82.2%)		
CPAP	54 (28.1%)	7 (15.6%)		
Nasal Cannula	5 (2.6%)	1 (2.2%)		
Diagnosis			1.72	0.423
RDS	189 (98.4%)	44 (97.8%)		
TTN	1 (0.5%)	1 (2.2%)		
Other	2 (1.0%)	0 (0.0%)		
Injury timing (day of life)			6.59	0.037
Within 1 week	148 (77.1%)	28 (62.2%)		
1–2 weeks	24 (12.5%)	6 (13.3%)		
≥3 weeks	20 (10.4%)	11 (24.4%)		

Data are presented as mean ± SD, median (IQR), or n (%). Comparisons between groups used *t*-test, Mann–Whitney U, or χ^2^ test as appropriate. PEEP: positive end-expiratory pressure; FiO_2_: fraction of inspired oxygen; NIV: non-invasive ventilation; CPAP: continuous positive airway pressure; NIPPV: nasal intermittent positive pressure ventilation; RDS: respiratory distress syndrome; TTN: transient tachypnoea of the newborn.

**Table 3:** Forward stepwise logistic regression analysis for predictors of moderate–severe nasal injury.

Variable	Category (Ref = No)	aOR	95% CI	*p*-Value
Previous injury	Yes vs. No	6.253	1.106–35.347	0.038
Injury location	Septum/Both vs. Bridge	2.977	1.036–8.429	0.043
Duration of PPV at delivery (min)	Yes (longer) vs. No	1.227	1.042–1.445	0.014

Forward stepwise logistic regression. Odds ratios (OR) with 95% confidence intervals are reported. PPV: positive pressure ventilation.

**Table 4:** Respiratory characteristics and outcomes of infants with NIV-associated nasal injury by timing of injury (≤3 vs. >3 days of device use).

Variable	≤3 Days (n = 169)	>3 Days (n = 68)	Odds Ratio/Mean Difference (95% CI)	*t*/Z/χ^2^	*p*-Value
Respiratory support				5.25	0.072
Nasal cannula	2 (1.2)	4 (5.9)			
CPAP	47 (27.8)	14 (20.6)			
NIPPV	120 (71.0)	50 (73.5)			
Previous injury	2 (1.2)	10 (14.7)	14.39 (3.06–67.64)	18.44	<0.001
PPV at birth	95 (56.2)	42 (61.8)	1.26 (0.71–2.24)	0.61	0.434
Postnatal steroids before injury	1 (0.6)	7 (10.3)	19.3 (2.32–159.9)	14	<0.001
Vasopressor before injury	3 (1.8)	1 (1.5)	0.83 (0.08–8.08)	0.03	1.000
PEEP (cmH_2_O)	6.0 (6–7)	6.0 (5–7)		−0.55	0.583
FiO_2_ at injury (%)	0.273 ± 0.086	0.244 ± 0.050	0.03 (0.01–0.05)	2.56	0.006
FiO_2_ ≥ 0.30	32 (18.9)	4 (6.0)	0.27 (0.09–0.80)	6.24	0.013
Hemoglobin (g/dL)	15.7 ± 2.9	13.7 ± 2.9	2.03 (1.20–2.85)	4.84	<0.001
Lactate (mmol/L)	1.5 (1.1–2.0)	1.1 (0.8–1.4)		−5.48	<0.001
Base deficit/excess	−2.41 ± 3.24	0.16 ± 3.75	−2.58 (−3.54 to −1.61)	−5.26	<0.001
Duration of PPV (min)	3.1 ± 2.6	3.9 ± 3.5	−0.76 (−1.87 to 0.34)	−1.23	0.111
Duration of intubation before injury (days)	0.0 (0–2.0)	0.0 (0–7.5)		−3.35	<0.001
Duration of NIV before injury (days)	1.3 ± 0.82	9.5 ± 8.1	−8.22 (−10.18 to −6.26)	−8.36	<0.001
Severity stage ≥ 2	30 (17.8)	15 (22.1)	1.29 (0.64–2.60)	1.72	0.423
Injury location				17.28	<0.001
Bridge	135 (79.9)	38 (55.9)			
Septum/Columella	27 (16.0)	28 (41.2)			
Both	7 (4.1)	2 (2.9)			
Interface type				5.73	0.057
Mask	155 (91.7)	55 (80.9)			
Prongs	9 (5.3)	9 (13.2)			
Alternating	5 (3.0)	4 (5.9)			
Injury day-of-life (days)	3.3 ± 4.9	16.8 ± 17.8	−13.5 (−17.9 to −9.1)	−9.05	<0.001
PEEP category (cmH_2_O)				3.777	0.151
≤5	30 (18.0%)	17 (26.6%)			
6–7	130 (77.8%)	42 (65.6%)			
≥8	7 (4.2%)	5 (7.8%)			
Primary respiratory diagnosis				1.249	0.536
RDS	166 (98.2%)	67 (98.5%)			
TTN	2 (1.2%)	0 (0.0%)			
Other	1 (0.6%)	1 (1.5%)			
Intubation before injury	83 (49.1%)	45 (66.2%)	2.027 (1.128–3.642)	5.684	0.017
Received NIV before intubation	14 (8.3%)	9 (13.2%)	1.689 (0.694–4.110)	1.357	0.244
Injury day-of-life (day)				69.038	<0.001
<7 days	150 (88.8%)	26 (38.2%)			
7–14 days	13 (7.7%)	17 (25.0%)			
≥14 days	6 (3.6%)	25 (36.8%)			

Values are presented as numbers (percentage) or mean ± SD (or median [IQR]). Statistical comparisons were performed using Student’s *t*-test, Mann–Whitney U test, or χ^2^ test, as appropriate. CPAP: continuous positive airway pressure; NIPPV: noninvasive positive pressure ventilation; PPV: positive pressure ventilation; PEEP: positive end-expiratory pressure; FiO_2_: fraction of inspired oxygen; RDS: respiratory distress syndrome; TTN: transient tachypnoea of the newborn; NIV: noninvasive ventilation; CI: confidence interval; IQR: interquartile range.

## Data Availability

The data presented in this study are available on request from the corresponding author. The data are not publicly available due to privacy or ethical restrictions. Data requests should be made to Ashraf Gad at agad2@hamad.qa.

## Abbreviations

The following abbreviations are used in this manuscript:

AGA	Appropriate for Gestational Age
aOR	Adjusted Odds Ratio
BW	Birth Weight
CI	Confidence Interval
CPAP	Continuous Positive Airway Pressure
DOL	Day of Life
FiO_2_	Fraction of Inspired Oxygen
IRB	Institutional Review Board
IQR	Interquartile Range
LSCS	Lower Segment Cesarean Section
LGA	Large for Gestational Age
MRC	Medical Research Center
NC	Nasal Cannula
NIPPV	Nasal Intermittent Positive Pressure Ventilation
NIV	Noninvasive Ventilation
NICU	Neonatal Intensive Care Unit
NVD	Normal Vaginal Delivery
NY	New York
PEEP	Positive End-Expiratory Pressure
PPV	Positive Pressure Ventilation
RDS	Respiratory Distress Syndrome
SGA	Small for Gestational Age
SPSS	Statistical Package for the Social Sciences
TTN	Transient Tachypnea of the Newborn
SD	Standard Deviation
WWRC	Women’s Wellness and Research Center
